# Determining the accuracy of the leukocyte esterase reagent strip test in the rapid diagnosis of adult septic arthritis

**DOI:** 10.1186/s42358-024-00409-4

**Published:** 2024-08-30

**Authors:** Peyman Mirghaderi, Mohammad-Taha Pahlevan-Fallahy, Jamil Mahmoudi, S.M. Javad Mortazavi

**Affiliations:** 1 Surgical Research Society (SRS), Students’ Scientific Research Center, Tehran University of Medical Sciences, Tehran, Iran; 2 Joint Reconstruction Research Center, Imam Khomeini Hospital, Tehran, Iran

**Keywords:** Septic arthritis, Leukocyte esterase, Joint infection, Urine strip test, Diagnostic test, Acute hot joint

## Abstract

**Backgrounds:**

Septic arthritis is a dangerous disease that occurs when microorganisms enter synovial fluid. It needs fast and accurate management; otherwise, it can harm the patient’s life. Currently, the tests measure WBC and PMN in SF, so we hypothesized to use a proxy that is easier and faster to measure. Leukocyte esterase is an enzyme secreted by neutrophils that can be found in the synovial fluid of SA patients. In this study, we tried to investigate the sensitivity and specificity of leukocyte esterase in diagnosing septic arthritis.

**Methods:**

We obtained synovial fluid samples from forty-six patients suspected of having septic arthritis and fifty-eight healthy individuals and measured the WBCs, ESR, CRP, PMN, glucose, and protein of SF in 2021. We also used the leukocyte esterase dipstick test to investigate the level of LE in synovial fluid for one minute.

**Results:**

Based on clinical and paraclinical criteria, sixteen out of the forty-six patients were diagnosed with SA. When (++) was considered positive, the sensitivity and specificity of the LE dipstick test for the diagnosis of SA were 93.7% (95% CI: 81.8–100%) and 60% (95% CI: 42.4–77.5%, *P* = 0.000), respectively. When both (+) and (++) were considered positive, they were 100% and 43.3% (95% CI: 25.6–61.0% *P* = 0.000), respectively. All the patients in the control group had negative cultures and LE test readings (specificity = 100%).

**Conclusion:**

The LE dipstick test can be a valuable diagnostic tool in the initial diagnosis of SA since it is affordable, fast, and reliable.

## Background

Septic arthritis (SA) is an acute condition in which an organism infects the synovial fluid (SF), mostly monoarticular. Various microorganisms can cause SA, with bacteria being the most common organism infecting SF and *S. aureus* being the most prevalent [1, 2]. Nearly 20,000 cases of SA occur annually in the US, accounting for 7.8 cases in 100,000 person-years [3]. In 2012, SA accounted for 16,382 emergency department (ED) visits, which comprised 0.01% of ED visits and cost $36.9 m [4]. The incidence is higher in rheumatoid arthritis (RA) patients, as they are more susceptible to SA, use immunosuppressive drugs and have a preexisting joint disease [1, 5]. It is essential that SA is diagnosed and treated as soon as possible. Otherwise, it might destroy the joint, cause severe degeneration, and impair the patient’s life for a long time. In extreme cases, late or incomplete treatment might lead to subcartilaginous bone destruction, cartilage damage, joint dysfunction, reinfection (14.3%), and death (2.8–11%) [6–8]. A study estimated that in people older than thirty, septic arthritis patients (1.29%, 95% CI = 1.03–1.60) are six times more prone than the normal population (0.21% 95% CI = 0.21–0.21) to requiring arthroplasty [7].

The gold standard test for detecting SA is SF culture. SF culture is time-consuming and relatively expensive, and Gram staining does not yield the most accurate results (sensitivity: 27–81%, specificity: 99–100%) [9]. The SA management guidelines suggest that SF and blood samples be sent for analysis and culture as soon as possible. Empiric antibiotic therapy is initiated immediately after waiting for culture. Gram stain might take days and waste the opportunity to prevent the disease from exacerbating and preventing joint destruction.

Various diagnostic tests exist for detecting SA and ruling out other possible differential diagnoses for monoarthritis, which require entirely different treatment approaches (gout and pseudogout, reactive arthritis, rheumatoid arthritis, and acute traumatic arthritis, for instance). These tests are limited in sensitivity, specificity, or even both. C-reactive protein (CRP), erythrocyte sedimentation rate (ESR), white blood cell (WBC), procalcitonin (PCT) in serum and WBC, polymorphonuclear cell percentage (PMN%), lactate, glucose, and PCT in the SF are some examples of diagnostic tests [9]. Other novel tests have also been investigated to help with the early detection of SA. Lenski and Scherer assessed the accuracy of detecting SA using SF total protein (sensitivity: 49–59%, specificity: 67–75%), lactate dehydrogenase (LDH) (sensitivity: 66–69%, specificity: 73–89%), uric acid (sensitivity: 78%, specificity: 83%) and IL-6 (sensitivity: 93% specificity: 64%) [10–12]. Omar et al. assessed the accuracy of the leukocyte esterase (LE) dipstick strip test using SF (sensitivity: 95%, specificity: 73%) [13].

Leukocyte esterase is an enzyme that breaks down esters; neutrophils produce it, and when they are recruited to or damaged sites, they can be detected in the infection site. The LE dipstick test is an inexpensive and fast diagnostic method widely used to detect urinary tract infections (UTIs). Additionally, a meta-analysis has proven its applicability for diagnosing spontaneous bacterial peritonitis (SBP) in cirrhotic patients [14]. It has also been proven helpful in diagnosing exudative pleural effusion (sensitivity 93.1%, specificity 50%) [15]. Its accuracy in diagnosing SA has recently attracted attention. In a meta-analysis by Dey et al. comparing the sensitivity and specificity of different diagnostic methods of SA, LE levels in SF were investigated in four studies (sensitivity: 0.94 (95% CI: 0.70–0.99), specificity 0.74 (95% CI: 0.67–0.81)) [16]. If the LE dipstick test proves to be a valid diagnostic technique, it is advantageous since it is inexpensive, quick, and easy to administer.

This study aimed to investigate the accuracy of the LE dipstick test in the early diagnosis of SA.

## Materials and methods

### Study design, setting, and participants

This prospective cross-sectional study is a diagnostic accuracy test following the STARD 2015 protocol [17]. It includes all patients who visited our tertiary referral center (*Blinded hospital, city, and country*) in 2021. Patients aged more than 18 were included in this study. Patients with a history of antibiotic treatment in the past two weeks before their admission or patients whose SF sample contained too much blood that did not allow for an accurate reading (according to the manufacturer, the test result would still be correct if there were less than 10,000 RBCs/µL in the sample) or whose aspiration volume was insufficient (less than a drop) were excluded from this study.

Patients with acute (24–48 h) signs and symptoms that were alarming of septic arthritis (two out of pain, warmth, fever, decreased range of movement, and redness, or effusion/swelling on its own) were assessed by a physician. The study participants consisted of two groups of people. First, patients with a monoarticular inflammatory complaint in one of their knees clinically suspected of SA were included in the study as group A. patients with other confirmed diagnoses such as spondyloarthropathies, gout, rheumatoid arthritis, and patients with a K-L grading >1 were excluded from the study. Group B consisted of controls who needed joint fluid aspiration as part of their medical care before knee arthroplasty but showed no sign of septic arthritis.

### Definitions

Patients were suspected of SA if they met the following criteria and were diagnosed with SA if they had a positive synovial fluid culture as the gold standard test; sudden severe monoarticular tenderness, effusion/swelling, decreased range of motion, warmth, redness, limping along with fever, and visible pus in the joint fluid.

### Data gathering

A trained specialist collected all synovial fluid samples before any surgery or treatment plan with a syringe size of 18–22 for SF aspiration in a sterile environment. SF samples were divided into three containers; the first was sent for laboratory analysis (LDH, protein, glucose, WBC), the second was designated for culture, and the third was assigned for LE dipstick strip test; the first and the second containers were sent to the labs for analysis and culture within thirty minutes. Urine LE dipsticks (Chemstrip 7; Roche Diagnostics, Indianapolis, Indiana, USA) were used to assess LE in the third container. This strip contains seven reagent pads to measure blood, proteins, glucose, nitrates, pH, ketones, and leukocyte esterase. In this study, we only focus on the LE test. The color change in the LE reagent pad occurs when indoxyl carbonic acid ester is hydrolyzed to indoxyl by the LE enzyme, which is derived from live or lysed leukocytes in the synovial fluid. The product reacts with a diazonium salt and produces a color that the user reads. There are four possible readings: white (negative), a faint purple color (trace), and light and dark purple, which are + and ++, respectively.

Each container was tested twice with two LE strips by the same person, and the results were recorded one minute after the strip was inserted into the container. According to the producer’s instructions, if a test yielded trace results, the user waited another minute, then reread the strip and recorded that result as the final reading. In case the two readings were different, the lowest one was considered.

Finally, the results (negative, trace, +, ++) were used for statistical analysis. A final trace reading was always considered negative because of its high rate of false positive [18]. In the final analysis, borderline results (+) were once considered to be negative, only (++) results were considered positive, and once both (+) and (++) results were positive.

### Sample size

Recent studies have estimated the sensitivity of using the leukocyte esterase dipstick test for the early detection of SA to be nearly 80% [13, 19–22]. Since synovial fluid aspiration and culture is the gold standard test, its sensitivity was considered 1. The highest acceptable type I and II errors were considered 0.05 and 0.2, respectively. The minimum sample size was then calculated to be 40.

$$n=2\frac{{{{({z_{1 - \frac{\alpha }{2}}}+{z_{1 - \beta }})}^2}\bar {p}\bar {q}}}{{{{({p_1} - {p_2})}^2}}}$$

### Statistical analysis

The LE dipstick test was assessed for its diagnostic accuracy, including sensitivity, specificity, positive predictive value (PPV), negative predictive value (NPV), positive likelihood ratio (PLR), and negative likelihood ratio (NLR), which were compared with the gold standard method (positive culture or visible pus). The final results were reported along with their standard error (SE) and 95% confidence interval (CI), which were calculated using the binominal distribution method. Student’s t test was used to compare means between groups for quantitative data analysis, and Pearson’s chi-square and Fisher’s exact tests were used to compare qualitative data. *McNemar’s* test was used to compare the diagnostic accuracy results between the gold standard methods and the LE test. SPSS version 25 was used for data analysis, and a *P* value < 0.05 was considered significant.

## Results

### Study population and demographics

Based on the inclusion criteria, 46 patients in Group A and 58 subjects in Group B were finally included. In group B, the controls, all of the cultures and leukocyte esterase readings were negative; also, no pus was observed in the joint fluid aspiration. This shows a specificity of 100% in the control population. In group A, 46 patients were analyzed, and sixteen (34.7%) participants were diagnosed with SA which estimates the pretest probability of SA in our study sample to be 34.7%. Their ages ranged from 18 to 86, with a mean of 43.5 (95% CI: 25.3–61.7) years. Twenty-six (56.5%) group A participants were males, and 20 (43.5%) were females. The patients had a body mass index of 28.3 ± 1.8 (28.4 ± 1.9 in patients and 28.2 ± 1.8 in controls, *P* = 0.24) All examined joints were knees.

Seven (15%) group A patients had positive SF smears, all with ++ LE test readings. Three (6%) had positive blood cultures, and all of them had ++ LE test readings. Sixteen (46%) had positive SF cultures, of whom 15 had ++ and one had + LE test readings.

Thirty-eight (82%) patients had high ESR (>20 mm/h), of whom 25 had ++ and 5 had + LE test readings. Ten (21%) had fever (>38.5 °C), of which eight had ++ and two had + readings. Twenty-eight (60%) had high blood WBC counts (>12000 cells/µL), of which 19 had ++ and one had + LE readings.

A summary of the lab findings of group A can be seen in Table 1.

**Table 1:** Summary of lab findings in the study

Factor measured	Min	Max	Mean (SD)
Age	18	80	43.5 (18.2)
ESR (mm/h)	5	125	57 (34)
CRP (mg/L)	1	108	48.2 (30.6)
Blood WBC (per µL)	1600	25,000	12,272 (5314)
Blood PMN %	22	92	75.4 (13.6)
Blood Lymphocyte %	10	80	25.8 (15.1)
SF WBC (per µL)	1600	25,000	12,273 (53.4)
SF PMN %	50	98	79.5 (9.3)
SF Lymphocyte %	2	50	21.5 (9.5)
Glucose in SF (mg/dl)	3	123	53.4 (25)
Protein in SF (mg/dl)	2	60	4.45 (0.8)

### Diagnostic accuracy results

The LE test was positive in 33 participants (71.7%) and negative in 19 cases (38.7%). Twenty-seven (58%) patients had (++) and 6 (13%) had (+) LE test results.

When only (++) was considered positive, the sensitivity, specificity, and accuracy of the LE test for the detection of SA were 93.8% (95% CI: 81.8–100%), 60% (95% CI: 42.4–77.5%), and 71.7% (95% CI: 58.5–84.9%), respectively. The PPV and NPV were estimated to be 55.6 (95% CI: 36.8–74.2%) and 94.7% (95% CI: 84.6–100%), respectively. The positive likelihood ratio (PLR) and negative likelihood ratio (NLR) were also calculated to be 2.34 (95% CI: 1.48–3.69) and 0.10 (95% CI: 0.01–0.71), respectively. According to McNemar’s test, the ++ LE test reading had a significant difference from the golden standard methods (*P* = 0.003).

**Fig. 1:**
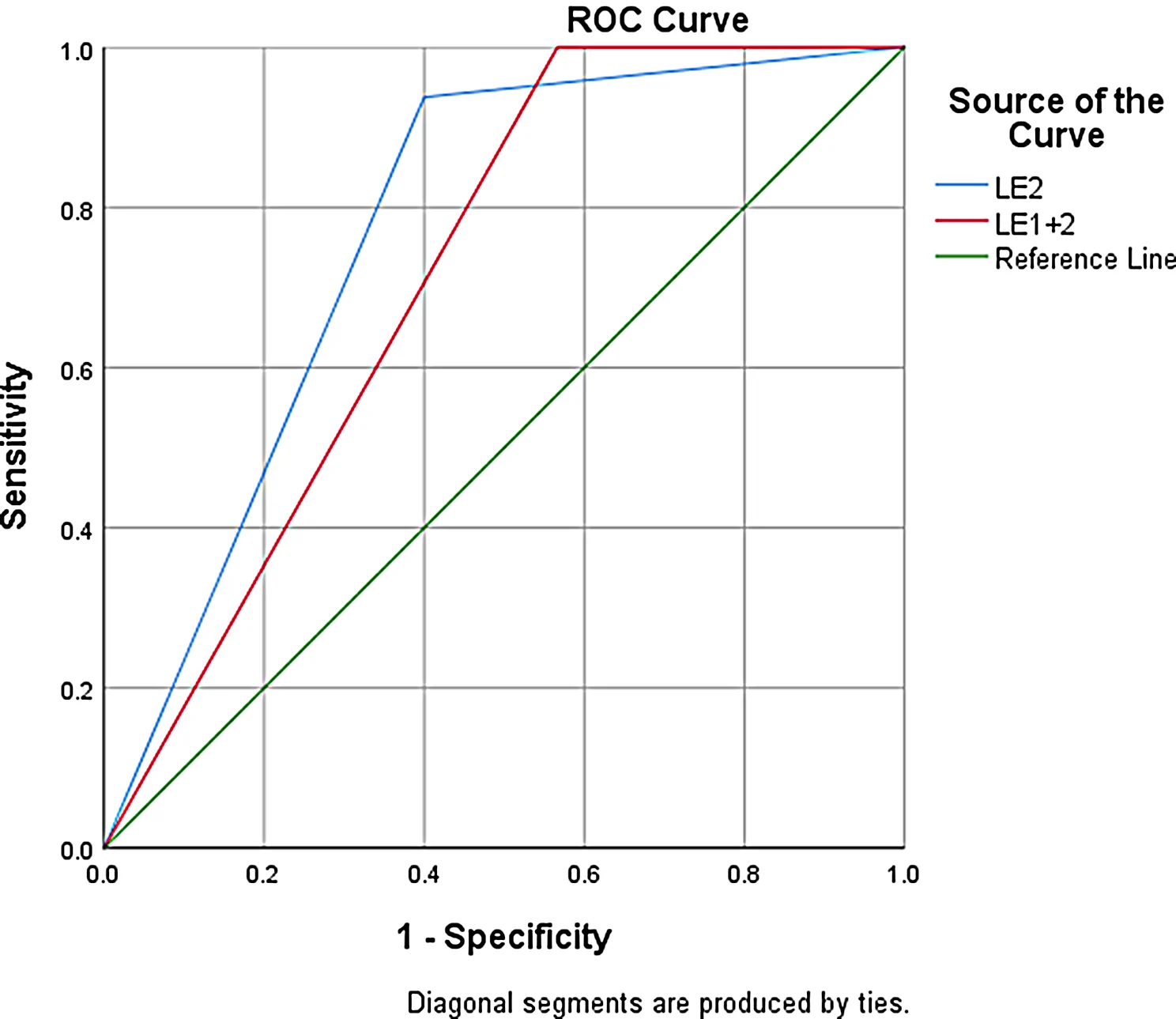
ROC analysis for LE test diagnostic accuracy (AUC = 0.72, 0.77 for +, ++ and ++, respectively)

When (+) and (++) were both considered positive, the sensitivity and specificity and accuracy of the LE test for the detection of SA were 100% and 43.33% (95% CI: 25.6–61.0%) and 63.04% (95% CI: 48.9–77.2%), respectively. The PPV and NPV were also calculated to be 48.48 (95% CI: 31.4–65.5%) and 100%, respectively. The PLR and NLR were estimated to be 1.76 (95% CI: 1.29–2.41) and 0, respectively. According to McNemar’s test, + and ++ LE readings had a significant difference from the gold standard method (*P* = 0.000). With an area under the curve (AUC) of 0.72 (95% CI: 0.57–0.86) for +, ++ and AUC of 0.77 (95% CI: 0.63–0.90), the diagnostic accuracy can be considered good [23] (Fig. 1).

A summary of the diagnostic accuracy (sensitivity, specificity, PPV, NPV, accuracy) of the LE dipstick test can be found in Table 2.

**Table 2:** Summary of the diagnostic value of the LE dipstick test (± SE)

LE Results	(++)	(+, ++)
Specificity (%)	60 ± 8.7%	43.33 ± 8.8%
Sensitivity (%)	93.8 ± 5.9%	100 ± 0%
PPV (%)	55.6 ± 9.3%	48.48 ± 8.5%
NPV (%)	94.7 ± 5.0%	100 ± 0%
Accuracy (%)	71.7 ± 6.6%	63.04 ± 7.1%
LR+	2.345	1.76
LR−	0.10	0

## Discussion

In our study, the LE test had acceptable sensitivity and specificity for SA detection. When (+) and (++) were both considered positive, the sensitivity and specificity and accuracy of the LE test for the detection of SA were 100% and 43.33% (95% CI: 25.6–61.0%) and 63.04% (95% CI: 48.9–77.2%), respectively. When only (++) was considered positive, the sensitivity, specificity, and accuracy of the LE test for the detection of SA were 93.8% (95% CI: 81.8–100%), 60% (95% CI: 42.4–77.5%), and 71.7% (95% CI: 58.5–84.9%), respectively. Our results show that with an AUC of 0.77, LE test can be a useful diagnostic method when used along with SF culture to provide a fast and sensitive diagnostic method for clinical decision making in the time it takes for culture results.

Diagnosing SA can be challenging since various differential diagnoses can manifest as SA. Patients typically do not present with the typical signs of infection. These vague clinical manifestations can mislead physicians to other differential diagnoses, such as Perthes’s disease or juvenile idiopathic arthritis (JIA) [16]. The current clinical and laboratory tests for SA are either time-consuming or do not have high sensitivity and specificity. In 1976, Newman proposed a set of diagnostic criteria to diagnose bacterial arthritis, including whether any organism was isolated from the joint, from blood, or histological or radiological findings of infection even in the case of no organism isolated or turbid fluid aspiration [24]. Currently, the most reliable methods to diagnose SA are SF aspiration studies, such as smear and culture. Various laboratory markers aid clinicians in diagnosis and can be used in conjunction with SF culture studies.

ESR and CRP are two of the most commonly used laboratory tests for SA diagnosis. Kocher’s criteria, being the most accepted diagnostic criteria for diagnosing SA in children, use WBC count > 12,000 cells/mm^3^, ESR > 40 mm/hour, fever > 38.5 °C, and whether the limb is weight bearing or not. In case one criterion exists, there is a 3% chance of SA; in the presence of two, 40%; in the presence of three criteria, 93%; and in case all four criteria are positive, there is a 99% chance of SA [25]. The sensitivity and specificity of serum ESR have been estimated to be between 34% and 100% and 23% and 93%, respectively [9, 26–29]. Serum CRP has been widely studied as a diagnostic marker for SA. Its sensitivity and specificity vary in different studies depending on the cut point between 58% and 100% and 0% and 96%, respectively [9, 12, 26, 28–32]. Although used in Kocher’s criteria, the serum WBC count is not a very sensitive marker since its sensitivity and specificity range between 20 and 62% and 61–100%, respectively [9, 12, 27–32]. SF WBC counts with a cutoff point of 50,000 cells/mm^3^ have a sensitivity of 54–100% and a specificity of 66–97% [9, 27, 28, 30, 31]. Procalcitonin (PCT), a relatively novel marker, can be measured in serum and SF. In SF, it has a sensitivity of 17–87% and a specificity of 55–100% [9, 27–30]. In serum, it has shown a sensitivity of 80–100% and specificity of 68–100% [9, 28, 29, 33]. There are many other markers, such as SF PMN%, glucose, LDH, calprotectin, IL-6, uric acid, lactate, serum IL-6, TNF-alpha, uric acid, and many others, which can be utilized together with other diagnostic tools [9].

Since SA is a debilitating disease that needs timely management, a late or underdiagnosis is unacceptable in the case of SA. Thus, a sensitive and accurate diagnostic test is paramount. This study investigated the LE strip test to potentially improve the orthopedic surgeon’s diagnostic ability regarding timeliness and accuracy. The LE dipstick test is relatively fast and affordable for detecting leukocytes in body fluids. By detecting the presence of the esterase enzyme produced by leukocytes, it was previously used for detecting UTIs and other infections.

Some studies have previously investigated the role of the LE dipstick test in diagnosing septic arthritis (Table 3). Knapper et al. investigated the diagnostic accuracy of the LE test in eighty adult patients (74% men, 26% women) in three hospital sites between 2015 and 2016. LE results were reported as negative (*n* = 9, 11%), + (*n* = 14, 18%), ++ (*n* = 24, 30%), and +++ (*n* = 33, 41%). Negative and trace (+) results were considered negative, and 57 patients had positive (++, +++) LE results. Five patients had positive 48-hour cultures. Of the remaining 52, 34 were diagnosed with crystal arthropathy, 17 were presumed to have an arthritic flare, and no cause was identified in one case. Their study yielded the results of 100% sensitivity, 30.7% specificity, 8.77% PPV, and 100% NPV [34]. A summary of the diagnostic accuracy of LE in SA diagnosis can be seen in Table 3. LE’s sensitivity (ranging from 80 to 100%) and specificity (ranging from 30 to 100%) in diagnosing SA were high, even more than in some other tests. Therefore, due to its high sensitivity, it can be used as a valuable screening tool. As it can be seen in Table 3, in the studies conducted by Colvin et al. and Knapper et al., LE test has a sensitivity of 100% and in the study by Omar et al. and Kolbeck et al. the sensitivity of LE test is around 95% which combined with the high specificity of in the seven out of the eight similar studies, shows that LE test can be used in conjunction with the other diagnostic methods to facilitate diagnosis and treatment, the differences between the diagnostic accuracy measures between studies can originate from different factors such as variability in LE reagent strips, their interpretation, the time needed before reading the strip, the prevalence of the condition in the population, other confounding factors and other diagnosis that can lead to the presence of WBC in the synovial fluid and thus yielding a positive result for the LE test, such as inflammatory arthritis diseases.

**Table 3:** Diagnostic accuracy of the LE dipstick test in recent studies

#	Study name	Sample size	Sensitivity	Specificity	PPV	NPV	Accuracy	PLR	NLR
1	Parvizi et al. [19]	186	80.6%	100.0%	100.0%	93.3%	N/A	N/A	0.19
2	Coiffier et al. [20]	98	79.2%	92.3%	96.6%	61.5%	N/A	10.3	0.23
3	Omar et al. [13]	146	94.7%	73.2%	34.6%	98.9%	N/A	114	0.11
4	Colvin et al. [21]	57	100.0%	97.0%	95.0%	100.0%	94.7%	33.30	0.00
5	Gautam 2017 [36]	27	79.2%	80.8%	61.8%	90.1%	96.3%	4.13	0.26
6	Knapper 2021 [34]	80	100.0%	30.7%	8.8%	100.0%	35.0%	1.44	0.00
7	Yeganeh 2020 [37]	68	80.8%	78.6%	70.0%	86.8%	N/A	3.78	0.24
8	Kolbeck 2021 [38]	455	95.0%	82.0%	47.0%	99.0%	84.3%	5.34	0.06

The LE test can be performed and returns a reliable result within a minute or two. This can also be useful when the clinician faces doubt regarding SA preoperatively. Parvizi et al. previously investigated the accuracy of the LE test in detecting the cause of periprosthetic joint infection (PJI) [19]. In the study conducted by Parvizi et al., a (++) reading on the LE test meant that the diagnosis of PJI was nearly confirmed, and a negative or trace result almost ruled out septic arthritis [19]. Based on the results of our study and other researchers’ studies, we can claim that the LE dipstick, either by itself or in conjunction with other diagnostic methods, can be used as a screening test for SA in suspected patients. Since LE is a product of leukocytes, it can be said to be a proxy for detecting WBCs in the SF. As Parvizi et al. claimed, detecting LE instead of WBC and measuring PMN percentage has a fascinating advantage in that it can include dead cells. In contrast, WBC measurement does not include dead cells in the case of very aggressive infections, and there are a high number of dead WBCs in the SF [19]. Therefore, the LE test can be used initially to screen SA before a more time-consuming and expensive workup is needed.

### Limitations

The LE test has some negative points. One limitation of the LE dipstick test is that it requires the collection of synovial fluid, which can be painful for patients since it is an invasive procedure. Additionally, the LE test is subject to false positives and negatives, which can lead to unnecessary treatment or delayed diagnosis. False positives can occur in patients with other inflammatory joint diseases, such as rheumatoid arthritis, gout, or reactive arthritis; in contrast, false negatives can occur in patients with septic arthritis who do not have leukocytosis or those who receive antibiotic treatment before the test [35].

Our study faced some limitations. First, the qualitative nature of LE test results might make it challenging to suggest a precise cutoff value of LE enzyme in SF to rule out or confirm SA diagnosis. Second, there are various protocols for administering LE, varying the time the dipstick is in the sample and the number of tries. Consequently, our protocol may differ from previous protocols, indicating heterogeneity between the studies. Conducting studies with larger sample sizes can help determine the exact diagnostic accuracy of the LE test. Also, since we could not indicate an aggressive procedure like joint fluid aspiration for healthy people our controls were patients undergoing knee arthroplasty and this makes our study susceptible to Berkson bias. The primary goal of our study was to assess the diagnostic accuracy of LE dipstick in early diagnosis of SA and we did not further gather for other diseases such as RA, AS, gout, etc. and excluded patients with any probable diagnosis other than SA from our study which would be valuable for future studies to inquire about.

## Conclusion

In conclusion, the LE dipstick test can be a useful tool in screening patients with suspected septic arthritis due to its sensitivity, rapid results, and affordability. However, it should be used in adjunction with other laboratory tests and clinical evaluations to confirm or rule out a definitive diagnosis. Moreover, the test has limitations and is subject to false positives, which should be considered when interpreting the results and cannot be used instead of SF culture.

## Data Availability

All data generated or analysed during this study are included in this published article [and its supplementary information files].

## Abbreviations

SA: Septic arthritis
SF: Synovial fluid
ED: Emergency department
CI: Confidence interval
CRP: C-reactive protein
ESR: Erythrocyte sedimentation rate
WBC: White blood cell
PCT: Procalcitonin
LDH: Lactate dehydrogenase
PMN: Polymorphonuclear cell
LE: Leukocyte esterase
UTI: Urinary tract infection
SBP: Spontaneous bacterial peritonitis
PPV: Positive predictive value
NPV: Negative predictive value
JIA: Juvenile idiopathic arthritis
SE: Standard error
PLR: Positive likelihood ratio
NLR: Negative likelihood ratio
